# Epidemiology of childhood Guillan-Barre syndrome in the north west of Iran

**DOI:** 10.1186/1471-2377-7-22

**Published:** 2007-08-05

**Authors:** Mohammad Barzegar, Saeed Dastgiri, Mohammad H Karegarmaher, Ali Varshochiani

**Affiliations:** 1 Department of Pediatrics, Tabriz University of Medical Sciences; 2 Research Centre for Infectious diseases and National Public Health Management Centre; 3 Department of Community and Family Medicine, Tabriz University of Medical Sciences

## Abstract

**Background and aims:**

This study was carried out to investigate the incidence, annual time trend and some epidemiological and clinical features of Guillain-Barre syndrome in children in the north west of Iran.

**Materials and methods:**

In this population-based cross sectional research, epidemiological and clinical features of 143 cases with Guillain-Barre syndrome between 2001 and 2006 were studied. The setting of the study was Tabriz Children Medical Centre, the major University-Hospital located in Tabriz city of the East Azarbaijan province covering whole region. Data collected included age, gender, chronological information, preceding events, functional grade of motor deficit.

**Results:**

The mean age (standard deviation) of subjects was 5.4 (3.6) years. The male/female ratio was 1.3. The average annual incidence rate was 2.27 per 100 000 population of 15 years children (CI95%: 1.9–2.6). The majority of cases occurred in March, July and November and the highest proportion of the syndrome was observed in winter (29 percent, P > 0.10).

**Conclusion:**

The results indicated that an unexpected high incidence of Guillain-Barre syndrome has occurred in 2003 in the region. We concluded that a monitoring and surveillance system for Guillain-Barre syndrome is essential to set up in this region.

## Background

Guillain-Barre syndrome is an autoimmune disorder of peripheral nervous system causing progressive weakness and areflexia. Since the marked decline in poliomyelitis incidence, the syndrome is now the most common cause of acute flaccid paralysis in many countries [1]. Epidemiologic studies have reported an annual incidence of 0.16–4 (mostly between 1–2) cases per 100 000 population from different countries [2-10]. Although the disease is considered to be sporadic without significant variation over time, some studies have shown annual and seasonal trends [5-8,11-13].

The aim of this study was to investigate the incidence, annual time trend and some epidemiological and clinical features of Guillain-Barre syndrome in children in the north west of Iran.

## Methods

East Azerbaijan is located in the northwest of Iran, a cold climate zone. It has a total area of 47 821 km^2^. According to the official census data, the total population of children 0–15 years was estimated 6465267 over the study period.

In this population-based cross sectional research, medical history and clinical features of 143 cases with Guillain-Barre syndrome between 2001 and 2006 were studied. The setting of the study was Tabriz Children Medical Centre, is the largest children medical center in the north-west area of Iran. This medical centre is a 200-bed acute care university hospital providing tertiary referral care for critically ill patients.

As part of World Health Organization's (WHO) certification process for polio eradication, Iran has been systematically registering children under 15 years old with acute flaccid paralysis since 1995. However, the local policy for acute flaccid paralysis (AFP) surveillance program is that all cases of AFP should be referred to Tabriz Children Medical Centre. All subjects are routinely examined by expert child neurologist (Mohammad Barzegar, the principal investigator of this project) within 7 days of notification. The GBS cases (under 16 years) were then diagnosed and aascertained based on the criteria defined and introduced by Asbury and Cornblath [1].

Data collected included age, gender, chronological information, preceding events, neurological features functional grade of motor deficit and laboratory findings. The functional status at the time of maximum deficit was graded according to Hughes scale of disability as follows: 0: healthy, 1: minor signs and symptoms and is capable of running; 2: able to walk 5 meters without assistance, but is unable to run. 3: able to walk with assistance, 4: confined to bed or chair bound, 5: requires assisted ventilation, and 6: died [14].

Poliovirus infection was excluded by cultures that are routinely performed for patients with acute flaccid paralysis as a requirement of the national program of poliomyelitis eradication.

All children underwent at least one electro diagnostic evaluation at the acute phase of disease (from day 1 to 27, average 6.5 days). A Medelec Synergy electromyography machine was used for this assessment. Nerve conduction studies included motor nerve conduction (MNC), sensory nerve conduction (SNC), and F-wave response studies were performed using the standard techniques of supramaximal percutaneous nerve stimulation and surface electrode recording. MNC studies were done on the ulnar, tibial and deep peroneal nerves and SNC on median and sural nerves. Each value of nerve conduction was compared with age matched normal data reported by Parano and colleagues [15]. Needle EMG was done for any denervation and motor unit action potential changes in all patients in at least two proximal and two distal limb muscles. Patients were classified as having axonal or demyelinating type based on the electrodiagnostic criteria reported by Cornblath and colleagues [16]. For each patient, the first neurophysiologic study was reviewed.

Approval for this study was obtained from National Public Health Management Centre of Tabriz University of Medical Sciences where a funded project is routinely assessed/approved in terms of methodology, ethical and financial issues.

Incidence rates and descriptive statistics were calculated to document the epidemiological features of the Guillain-Barre syndrome in the area. Data from the Ministry of Health, Statistics Office, were used to estimate the expected frequencies of the syndrome in the East Azarbaijan province to assess the time trend.

## Results

Between 2001 and 2006, one hundred forty three cases of Guillain-Barre syndrome were diagnosed and ascertained in Tabriz Children university-hospital of Tabriz University of Medical Sciences, East Azarbaijan province, Iran.

Table-1 shows the basic characteristics of the study subjects. The mean age (standard deviation) of cases was 5.4 (3.6) years (range: 1–15 years). The male/female ratio was 1.3. The functional grade of motor deficit was scored 4 in the majority of the cases (67.1%). Fifteen patients (10.5%) received assisted ventilation, and two (1.4%) died. In electrodiagnostic study, three patterns emerged: demyelinating type (53.8%), axonal type (35%), and 11.2% as normal.

**Table 1:** Basic and clinical characteristics of the study subjects with Guillain-Barre syndrome

		*Mean*	*Standard Deviation*
		
***Age (years)***		*5.4*	*3.6*
***Median time to peak disability(days)***		*4.7*	*3.7*
		
		*Number*	*Percent*
		
***Sex***	***Male***	*81*	*56.5*
	***Female***	*62*	*43.5*
***Preceding events ***			
	***Upper Respiratory infection***	*75*	*52.4*
	***Gastroenteritis***	*20*	*14*
	***other ***	*4*	*2.8*
	***None ***	*44*	*30.8*
***Seasonal incidence ***			
	***winter ***	*41*	*28.7*
	***spring***	*28*	*19.6*
	***summer ***	*38*	*26.6*
	***autumn***	*36*	*25.2*
***Functional Grading***	***2***	*8*	*5.6*
***of disease***	***3***	*24*	*16.8*
	***4***	*96*	*67.1*
	***5***	*13*	*9.1*
	***6***	*2*	*1.4*
***Cranial nerve Involvement***		*57*	*39.9*
***Autonomic dysfunction ***		*21*	*14.7*
***Sensory symptoms (limbs pain)***		*43*	*30.1*

Upper respiratory tract infections were most common preceding infection in winter (72.5%). Sensory symptoms, mostly as limbs pain were observed in 43 patients (30.1%). Cranial nerve involvement was also observed in about 40% of patients. Bulbar weakness (22%) was the most common type of cranial nerve involvement followed by facial palsy (17%).

The average annual incidence rate was 2.27 per 100 000 population of 15 years children (CI95%: 1.9–2.6) in the area ranging from 1.5 to 3.4 in different years.

**Figure 1:**
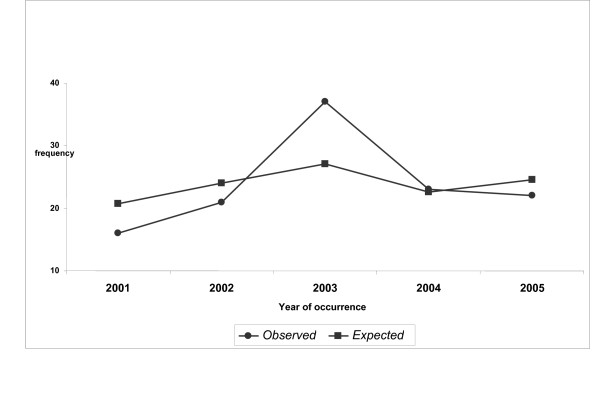
Observed and expected frequency of the Guillain-Barre syndrome in the north west of Iran.

There was a marginal significant variation in the trend in incidence rates (per 100 000 population of 15 years children) of Guillain-Barre syndrome between 2001 and 2006 indicating that an unexpected high incidence of syndrome has occurred in 2003 in the region (Table 2). The same trend was again observed for the year 2003 when the observed frequencies of the syndrome at the same region were compared to the expected values (Figure 1).

**Table 2:** Incidence of the Guillain-Barre syndrome in the north west of Iran

**Diagnosis year**	**Incidence (per 100 000)**	**95% Confidence Intervals**
**2001**	1.49	(0.8,2.4)
**2002**	1.95	(1.2,2.9)
**2003**	3.44	(2.4,4.7)
**2004**	2.14	(1.3,3.2)
**2005**	2.04	(1.2,3.1)
**2006**	2.57	(1.7,3.7)

**Figure 2:**
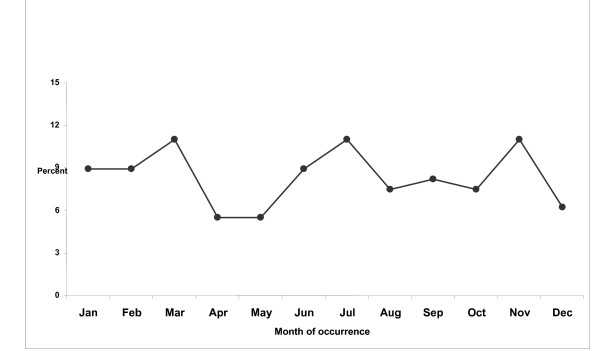
Occurrence of the Guillain-Barre syndrome in the north west of Iran by calendar months.

Figure 2 shows the occurrence of the Guillain-Barre syndrome in the north west of Iran by calendar months. The majority of cases occurred in March, July and November in the whole study period. The lowest and highest proportion of the syndrome occurred in spring (19.6 percent) and winter (28.7 percent), respectively (P > 0.10).

## Dsicussion

We investigated the incidence, annual time trend and occurrence of Guillain-Barre syndrome in children in the north west of Iran.

Tabriz Children Hospital is the reference inpatient center for child neurology in the north-west of Iran. Therefore it is unlikely that patients with suspected Guillain-Barre syndrome were not visited/diagnosed/ascertained at this medical centre. However it is possible that some cases may have been missed, especially those with minor clinical signs and symptoms (grade 1) not requiring hospitalization. In our research, clinical features and incidence rate were similar to those reported from other studies [2-6,8,10-13].

In our study, the average incidence rate was slightly higher than those reported from most areas in the world (within the range between 0.16 to 4 cases per 100 000). However if we exclude the cases from year 2003, our incidence rate decrease to 2 cases per 100 000 population which is consistent with other reports [5-8,11,12].

The highest incidence in the year 2003 could not be related to any pathogen agent as campylobacter jejuni is not routinely detected in our medical centre. However in an investigation carried out by authors from January 2003 to march 2005 in the same region, serological evidence of recent campylobacter jejuni infection was found in about half of children with Guillain-Barre syndrome [17].

Outbreaks of the disease have been reported from different areas in the last few decades. Investigators from Greece have reported an outbreak in year 2002 [5]. A similar feature was studied/reported in Sweden in 1985 and 1992 [18]. An increased incidence of Guillain-Barre syndrome in the USA for 1976 was attributed to 'swine flu" vaccines [19]. Another study from Caribbean island of Curacao showed that incidence rose sharply from 1.62 (per 100 000) between 1987 and 1991 to 3.10 (per 100 000) between 1992 and 1999 [20].

Although the disease is considered to be sporadic without significant variation between seasons and months, small clusters occurred in March, July and November and the highest proportion of the syndrome was observed in the winter. Small clusters have been associated with outbreaks of bacterial enteritis caused by contaminated water. A research report from China indicated that summer epidemics of the syndrome might be caused by campylobacter jejuni infection [21]. In a study from Saudi Arabia, analysis of seasonal incidence has also shown that 40% of the cases occurred in the cold seasons with the highest peak in February [12]. In our study, clustering of patients in winter could be related to the high frequency of upper respiratory infection during cold season.

The percentage of antecedent infectious disease in the subjects in our investigation was almost similar to the average proportions reported from previous studies [2-8,10-13].

In the time period of 2002–3, a similar high frequency of Guillain-Barre syndrome was reported from the whole country (including neighboring provinces of study area). In seeking to explain this pattern, possible impact of some environmental causal or influencing factors can not be ruled out. More studies are needed to investigate the etiology of this time pattern.

The epidemiology of GBS is not easy to investigate because of the difficulties in case definition and the absence of a reference standard diagnostic test. Furthermore, objective physiologic abnormalities of nerve dysfunction may be difficult to detect at early stages. The difference in the incidence rates reported from different regions might then be partly explained by case definition, the design and methodology of the investigations (i.e. study design, population based vs hospital based settings, case ascertainment, etc).

## Conclusion

In conclusion, this study showed an unexpected occurrence of Guillain-Barre syndrome in the area and the whole country for 2003 indicating the necessity of an epidemiological surveillance system in the region for proper intervention in possible outbreaks in the future.

## Competing interests

the authors have no financial or personal relationships with other people or organizations that could pose a conflict of interest in connection with the present work. Tabriz University of Medical Sciences supported the whole project.

## Authors' contributions

Mohammad Barzegar and Saeed Dastgiri designed this study and reviewed the data. Ali Varshochiani coordinated the data collection. Mohammad HK Maher and Saeed Dastgiri generated and analyzed the statistical data. All authors contributed to the writing of the paper.

## Pre-publication history

The pre-publication history for this paper can be accessed here:


